# Focal overexpression of insulin-like growth factor 2 by hepatocytes and cholangiocytes in viral liver cirrhosis

**DOI:** 10.1038/sj.bjc.6600777

**Published:** 2003-03-04

**Authors:** N Sedlaczek, A Hasilik, P Neuhaus, D Schuppan, H Herbst

**Affiliations:** 1 Institute of Pathology, University of Muenster, Muenster, D-48129, Germany; 2 Department of Gastroenterology and Hepatology, Klinikum Benjamin Franklin, Free University of Berlin, Berlin, Germany; 3 Institute of Physiological Chemistry, Philipps-University, Marburg, Germany; 4 Department of Surgery, Charitè, Campus Virchow-Klinikum, Humboldt-University, Berlin, Germany; 5 Department of Gastroenterology and Hepatology, Friedrich-Alexander-University, Erlangen, Germany

**Keywords:** carcinogenesis, cirrhosis, insulin-like growth factor-2, mannose 6-phosphate/insulin-like growth factor-2 receptor, hepatic stellate cells, hepatocytes

## Abstract

Insulin-like growth factor (IGF)-2 is overexpressed in hepatocellular carcinoma and accompanying dysplastic lesions. IGF-2 signalling is mediated through IGF-1 receptor (IGF-1R), while mannose 6-phosphate/insulin-like growth factor-2 receptor (M6P/IGF-2R) controls pericellular levels of free IGF-2. We studied, by *in situ* hybridisation and immunohistology, 18 liver specimens with cirrhosis of different aetiology without neoplastic or dysplastic lesions. Immunohistology was also performed for insulin receptor IGF-1R and IGF-binding proteins 3 and 4. High focal levels of IGF-2 RNA were found in some hepatocytes of all livers with HBV- or HCV-induced cirrhosis (*n*=10), but in only one of the cirrhoses with nonviral aetiology (*n*=8). IGF-2 was overexpressed in biliary duct epithelial cells in one case. Compared with noncirrhotic liver, all cirrhotic specimens showed reduced hepatocellular expression of M6P/IGF-2R protein, which contrasted with enhanced expression in perisinusoidal cells. Immunostaining for the other antigens did not reveal significant differences. Upregulation of IGF-2 in some hepatocytes may lead to high focal IGF-2 levels sufficient to saturate local IGF-2 binding capacities, and may result in an increased susceptibility to cellular dedifferentiation and, ultimately, liver cancer. Downregulation of hepatocellular M6P/IGF-2R and upregulation of IGF-2 seem to be early events in hepatocarcinogenesis prior to the appearance of morphologically distinct dysplastic lesions. Elevated focal IGF-2 transcript levels may therefore indicate an increased risk for hepatocellular and cholangiocellular carcinomas.

The primary liver malignancies, hepatocellular carcinoma (HCC) and cholangiocellular carcinoma are highly aggressive neoplasms with a poor prognosis. Worldwide, HCC is the seventh most common cancer, with the highest incidence in areas endemic for hepatitis B virus (HBV) (Sterneck, 2000). HCC is also observed in cirrhosis of other aetiology, such as alcoholic liver disease, metabolic disorders, and, particularly, hepatitis C virus (HCV)-infection. Among cirrhotics, carriers of HBV and HCV have an approximately 200-fold increased risk to develop HCC (Sterneck, 2000). The incidence of HCC is increasing in Western countries, mainly because of an increased incidence of hepatitis C (Caselmann *et al*, 1997).

Insulin-like growth factor (IGF)-2 is involved in the regulation of liver cell growth and metabolism. IGF-2 is structurally related to proinsulin, IGF-1, and relaxin. The mitogenic and antiapoptotic properties of both IGF peptides as well as differentiation-related signalling are mediated primarily through IGF-1 receptor (IGF-1R). IGF-2 is physiologically expressed at high levels in various human and rodent foetal tissues such as liver, kidney, and skeletal muscle. In contrast, it is downregulated or virtually absent in the corresponding adult organs. Elevated levels of IGF-2 are characteristic for tumours originating from tissues expressing high levels of IGF-2-RNA during foetal life such as nephroblastoma (Wilm's tumour) (Reik and Maher, 1997), rhabdomyosarcoma (Ross *et al*, 2000), and HCC (Fiorentino *et al*, 1994) as well as in overgrowth disorders such as Beckwith–Wiedemann syndrome (Smilinich *et al*, 1999). High levels of IGF-2 peptide were detected in primary HCC and dysplastic foci of HCC-bearing livers compared to uninvolved liver tissue. Overall, IGF-2-RNA expression correlated well with the levels of IGF-2 peptide (Cariani *et al*, 1990; Fiorentino *et al*, 1994) and with the immunohistologically assessed proliferative activity in HCC (Nardone *et al*, 1996). In HCC, large proportions of the IGF-2-mRNA were found as splice forms characteristic for foetal tissues (Cariani *et al*, 1988). An allelic imbalance of IGF-2 gene expression correlating with the loss of maternal imprinting patterns was also considered to have a role in hepatocarcinogenesis (Aihara *et al*, 1998; Schwienbacher *et al*, 2000).

In the space of Disse, at least two mechanisms regulate the amounts of free IGF-2: its association with specific soluble high-affinity binding proteins (IGFBPs) and several IGFBP-related proteins (IGFBPrP) as well as an additional cellular receptor, the mannose 6-phosphate/insulin-like growth factor-2 receptor (M6P/IGF-2R) (Wetterau *et al*, 1999). In contrast to monotremes and marsupials, the M6P/IGF-2R is imprinted and able to bind IGF-2 in therian mammals (Killian *et al*, 2000). This receptor has a low affinity to IGF-1 and does not bind insulin. Unlike the IGF-1R, the M6P/IGF-2R has no intrinsic tyrosine kinase activity and is not associated with proliferation (O'Dell and Day, 1998). It is identical with the cation-independent mannose-6-phosphate receptor and its luminal moiety comprises 15 domains that can bind distinct ligands such as various mannose 6-phosphate-containing glycoproteins as well as IGF-2. The M6P/IGF-2R is involved in the transport of mannose 6-phosphate-containing glycoprotein and other proteins mainly from the *trans*-Golgi network and from the cell surface, respectively, to the endosomal/lysosomal compartments (Braulke *et al*, 1987). The transported proteins are either lysosomal enzymes or their substrates that may be subjected to processing, such as latent TGF-*β*, or degradation, such as IGF-2 (De Souza *et al*, 1997). An impairment of the internalisation of IGF-2 in a tissue may contribute to increased levels of IGF-2. Loss of heterozygosity within the M6P/IGF-2R alleles was observed in HCC and accompanying dysplastic lesions (Yamada *et al*, 1997).

All of this suggests that both the upregulation of IGF-2 expression and the downmodulation of IGF-2 binding capacity may influence the relative amounts of free IGF-2 peptide available for binding to the IGF-1R in HCC. Few studies of IGF-2 and M6P/IGF-2R expression are available, all of which were performed on tissues with manifest HCC. It is, therefore, not clear whether these mechanisms are active prior or parallel to the development of HCC or represent late events in hepatocarcinogenesis. This has prompted us to use *in situ* hybridisation and immunohistology to assess cellular levels of IGF-2 transcripts and M6P/IGF-2R antibody staining patterns in cases of cirrhosis of different aetiologies without morphological evidence of premalignant or malignant changes. Additionally, we compared the immunostaining profiles of the liver tissues for insulin receptor (IR) and IGF-1R as well as for IGFBP-3, the most abundant IGFBP present in healthy liver (Wetterau *et al*, 1999), and IGFBP-4 in order to explore the possibility that these proteins may ameliorate focally elevated IGF-2 levels.

## MATERIALS AND METHODS

### Tissues

A total of 21 liver tissue samples were available, 18 of which were from patients undergoing orthotopic liver transplantation. Tissues were snap frozen and stored in liquid nitrogen immediately after explantation. In parallel, tissue samples were formalin-fixed and paraffin-embedded by routine procedures. The cirrhoses were clinically related to viral hepatitis B (*n*=6), C (*n*=3) or B/D coinfection (*n*=1), to alcoholic liver disease (*n*=4), to autoimmune hepatitis (*n*=2), primary biliary cirrhosis (*n*=1), and primary sclerosing cholangitis (*n*=1). Tissues with preserved lobular architecture were from an explant with vanishing bile duct syndrome, a liver that was not used for transplantation owing to severe steatosis, and, distant from the focal lesion, from a resection specimen with a Klatskin tumour. Informed consent was obtained prior to surgery. Preparation of slides and frozen sections (5 μm) was done as described (Herbst *et al*, 1997). Formalin-fixed, paraffin-embedded mature placenta served as control for immunohistology on paraffin sections.

### Preparation and labelling of probe

A plasmid containing a 700 bp human IGF-2 cDNA (Dull *et al*, 1984) was obtained from the American Type Culture Collection, Bethesda, MA, USA (no. 57482) and was inserted into the transcription vector pGEM1. Authenticity of the probe was verified by restriction endonuclease digestion and partial sequence analysis. Run-off transcription and ^35^S-labelling of RNA probes with [^35^S] UTP and [^35^S] CTP were performed as described previously (Herbst *et al*, 1997).

### *In situ* hybridisation and immunohistology

*In situ* hybridisation in combination with immunohistology was carried out as described (Herbst *et al*, 1997) with monoclonal antibodies specific for cytokeratins, vimentin, desmin, and smooth muscle *α*-actin (clones MNF116, V9, D33, and 1A4, respectively). The antibodies were visualised by the APAAP method (Cordell *et al*, 1984) with affinity-purified mouse polyclonal antibodies to rabbit IgG, rabbit antibodies to mouse IgG, APAAP complex at a dilution of 1 : 40, and new fuchsin as the alkaline phosphatase substrate. All immunohistological reagents were from DAKO, Hamburg, Germany. Sections were processed simultaneously using the same batches of probes and reagents. Hybridisation of tissues pretreated with *Micrococcus* nuclease verified the RNA nature of the hybridisation target.

### Immunohistology

Cryostat sections (5 *μ*m) were incubated with the M6P/IGF-2R-specific monoclonal antibody, 2C2 (Braulke *et al*, 1987), diluted 1 : 40, and visualised by the APAAP method (Cordell *et al*, 1984). Immunostaining of formalin-fixed, paraffin-embedded tissue sections for the insulin receptor *β*-subunit (monoclonal antibody clone CT-3; NeoMarkers, Fremont, CA, USA), the extracellular domain of the IGF-1R (goat antibody, cat. no. AF305; R&D Systems, Minneapolis, MN, USA), IGFBP-3 (monoclonal antibody clone 84728.111; R&D Systems, Minneapolis, MN, USA), and IGFBP-4 (goat antibody, cat. no. AF804; R&D Systems, Minneapolis, MN, USA) was performed with LSAB (Labelled StreptAvidin-Biotin) kits with alkaline phosphatase-conjugated streptavidin and with peroxidase-conjugated streptavidin on a DAKO Autostainer. When appropriate, mouse anti-goat immunglobulin antibody was used. All of the secondary reagents and the automated immunostaining machine were supplied by DAKO.

## RESULTS

### IGF-2

**Figure 1:**
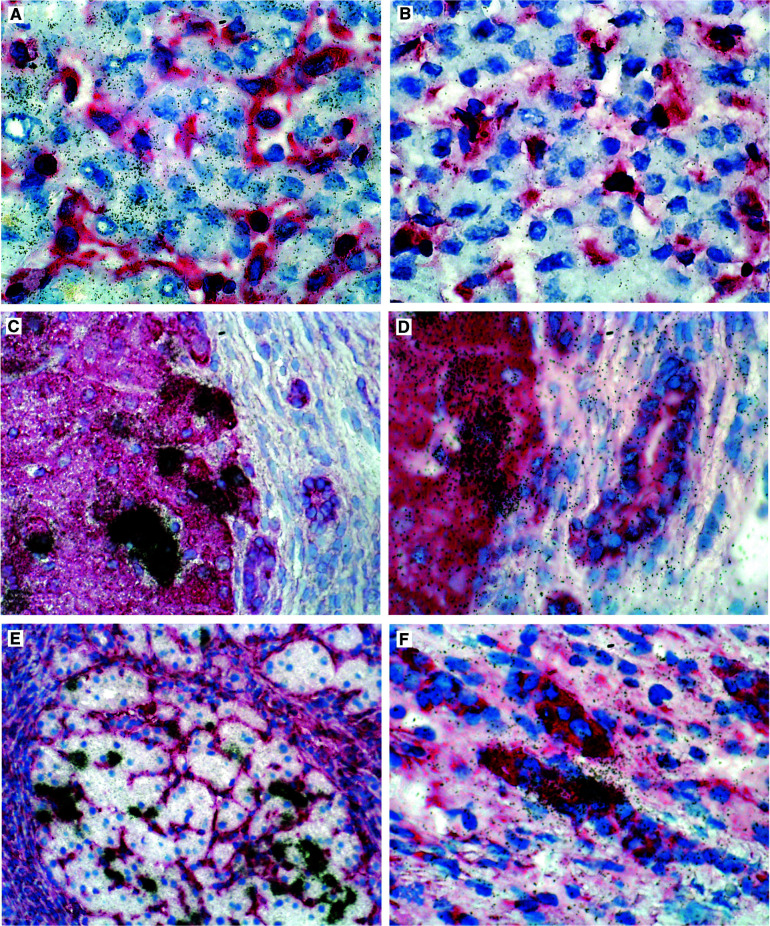
Expression patterns of IGF-2 in human normal and cirrhotic liver. Radioactive *in situ* hybridisation with single-stranded ^35^S-labelled antisense (**A**, **C**–**F**) and sense (control, **B**) IGF-2 RNA probes, combined with immunostaining for vimentin (**A**, **B**, **F**) or cytokeratin (**C**–**E**). Architecturally normal liver shows a homogeneous staining of hepatocytes, and vimentin-positive mesenchymal cells are negative (**A**). The sense probe does not produce a specific signal (**B**). Specimens with micronodular cirrhosis related to HBV infection (**C**–**F**) display IGF-2 RNA overexpression in clusters of cytokeratin-positive (**B,C**), vimentin-negative (**D**) hepatocytes. In one of the virus-associated cirrhoses, few cytokeratin-positive bile duct cells also overexpress IGF-2 transcripts (**F**).

IGF-2 was expressed in hepatocytes of all livers. In normal liver tissues, a homogeneously distributed weak labelling was observed among hepatocytes (Figure 1A

) without evidence of zonation. Bile duct epithelial cells displayed very low specific signals. Control hybridisations using the RNA probe in sense orientation produced a weak labelling homogeneously distributed over the tissue sections (Figure 1B). The pattern observed with normal liver samples was also found in specimens with cirrhosis of nonviral aetiology with one exception: one case with autoimmune hepatitis-related cirrhosis showed occasional groups of hepatocytes with moderately increased IGF-2-specific labelling. In contrast, all cases with viral aetiology (*n*=10) harboured small proportions of hepatocytes with dramatically increased IGF-2-specific signals (Figure 1C–E). These hepatocytes were assembled in clusters, usually at the periphery of regenerating nodules. In one case of HCV-related cirrhosis small groups of bile duct epithelial cells within fibrotic septa displayed significantly increased expression of IGF-2 as well (Figure 1F).

### M6P/IGF-2R

**Figure 2:**
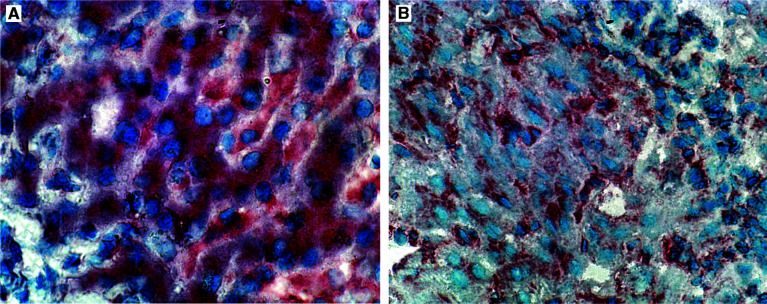
Immunostaining for the M6P/IGF-2R on normal (**A**) and cirrhotic liver (**B**, HCV-related cirrhosis) with the IGF-receptor-2 antibody 2C2. Normal liver displays cytoplasmic and membrane-specific immunostaining of hepatocytes (**A**). Cirrhosis related to HCV infection shows reduced immunostaining of hepatocytes restricted to the sinusoidal part of the cell membrane as well as increased signals in perisinusoidal cells (**B**).

In normal liver, the antibody 2C2 produced a diffuse cytoplasmic and membraneous staining of hepatocytes (Figure 2A

). In all of the cirrhotic livers, staining of hepatocytes was reduced in intensity and restricted to staining of the sinusoidal portions of the cell membrane. In contrast, a distinctly increased staining of spindle-shaped perisinusoidal mesenchymal cells was observed only in cirrhotic livers (Figure 2B).

### IR, IGF-1R, IGF-BP3, and IGFBP-4

**Figure 3:**
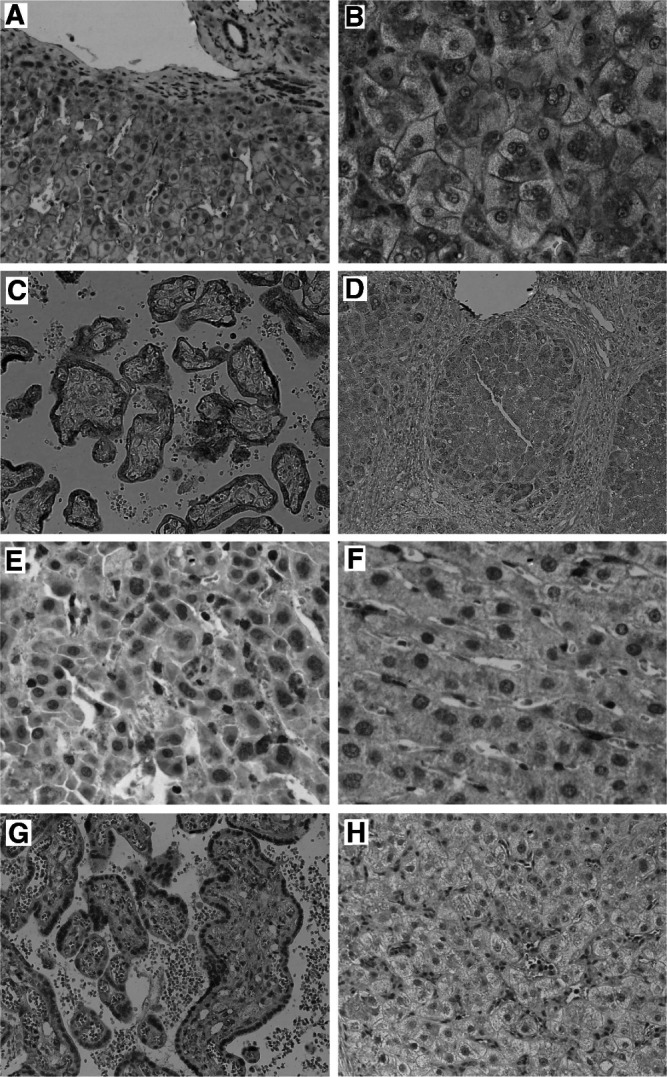
Immunostaining for the insulin receptor on normal (**A**) and cirrhotic liver (**B**) with monoclonal antibody CT-3, producing a membraneous staining without obvious differences between architecturally normal (**A**) and cirrhotic (**B**) liver. Immunostaining for the IGF-1R revealed a trophoblast epithelial staining pattern in mature placenta (**C**), and weak cytoplasmic and membraneous staining largely of hepatocytes in normal and cirrhotic liver (**D**). The IGFBP-3-specific monoclonal antibody reacted with sinusoidal cells on cirrhotic (**E**) and normal liver (**F**), part of which is morphologically compatible with Kupffer cells. Immunostaining for the IGFBP-4 on placenta for control (**G**) and cirrhotic liver (**H**) with the anti-human IGFBP-4 antibody. The IGFBP-4-specific antibody showed a clear cytoplasmatic staining in mature placenta (**G**), whereas cirrhotic liver displayed a weak immunoreactivity of cells also compatible with Kupffer cells.

Immunostaining for IR produced a membraneous staining without obvious differences between architecturally normal (Figure 3A

) and cirrhotic liver (Figure 3B), and without significant variation among the various cirrhotic specimens. Staining for the IGF-1R produced a clear epithelial staining in the mature placenta (Figure 3C) used for control. Independent of the detection system, we observed a weak cytoplasmic and membraneous staining in hepatocytes of all liver samples without differences between the various specimens (Figure 3D). Thus, IR and IGF-1R expression, as detected by immunohistology with the particular antibodies, neither displayed focal variation superimposable onto the expression pattern observed for IGF-2 nor showed significant differences between normal and cirrhotic liver. The IGFBP-3-specific monoclonal antibody specifically reacted with sinusoidal cells morphologically compatible with Kupffer cells/macrophages. The staining of Kupffer cells was variable among normal (Figure 3E) and cirrhotic (Figure 3F) livers, however, no distinct pattern emerged. IGFBP-4 showed a clear cytoplasmatic staining of stromal cells in mature placenta (Figure 3G) used for control. In all liver specimens with normal lobular architecture (Figure 3H) or cirrhosis, a weak immunostaining signal was found in sinusoidal cells. Also, the staining patterns did not reveal focal areas of increased signal intensity similar to the hepatocellular foci of IGF-2 RNA overexpression in viral cirrhosis. Table 1

**Table 1:** Expression patterns of IGF-2 and M6P/IGF-2R

			**IGF-2 RNA-pattern**			
**Case no.**	**Aetiology**	**Histology**	**HC diffuse**	**HC clusters**	**BDC**	**M6P/IGF-2R**
1	HBV	Cirrhosis	+	++++	0	+
2	HBV	Cirrhosis	+	++++	0	+
3	HBV	Cirrhosis	+	++++	0	+
4	HBV	Cirrhosis	+	++++	0	+
5	HBV	Cirrhosis	+	++++	0	+
6	HBV	Cirrhosis	+	++++	0	+
7	HBV/HDV	Cirrhosis	+	++++	0	+
8	HCV	Cirrhosis	+	++++	0	+
9	HCV	Cirrhosis	+	++++	++++	+
10	HCV	Cirrhosis	+	++++	0	+
11	AI	Cirrhosis	+	++	0	+
12	AI	Cirrhosis	+	0	0	+
13	Alc	Cirrhosis	+	0	0	+
14	Alc	Cirrhosis	+	0	0	+
15	Alc	Cirrhosis	+	0	0	+
16	Alc	Cirrhosis	+	0	0	+
17	PBC	Cirrhosis	+	0	0	+
18	PSC	Cirrhosis	+	0	0	+
19–21		Regular	0	0	0	++

AI=autoimmune hepatitis; Alc=alcohol-induced hepatitis; BDC=bile duct cells; HC=hepatocytes; PSC=primary sclerosing cholangitis; PBC=primary biliary cirrhosis; − and + to +++=average signal intensity relative to background labelling: −=background; +=1–2x; ++=2–5x; +++=5–10x; ++++=>10x background (background signal=5 five grains for BDC and 10 grains for HC).

## DISCUSSION

Comprising the growth factors, IGF-1 and -2, specific receptors, several binding proteins and binding protein-related proteins, the IGF system has an important role in diverse cellular functions including cell growth and survival. The liver is the major source of circulating IGF peptides. In contrast to foetal liver, IGF-2 is downregulated, and IGF-1 is upregulated in the adult organ (Scharf *et al*, 2001). IGF-2 gene expression is controlled by mechanisms such as parental imprinting resulting in downregulation of one allele. In HCC, loss of heterozygosity and changes in the methylation patterns within the IGF-2 genes have been documented, and the perturbation of these control mechanisms seems to result in upregulation of IGF-2 expression from promoters characteristic of foetal development (Aihara *et al*, 1998). Moreover, differential usage of foetal and adult IGF-2 promoters was observed in premalignant proliferations present in HCV-related chronic liver disease (Tanaka *et al*, 1996). The foetal promoters P2–P4 were active in HCC, whereas transcription from the adult promoter P1 was reduced in such lesions (Nardone *et al*, 1996; Li *et al*, 1997). Hepatic overexpression of the IGF-2 gene has been observed in animal models of hepatocarcinogeneses (Norstedt *et al*, 1988; Harris *et al*, 1998) as well as in human HCC (Sohda *et al*, 1996; Cariani *et al*, 1988), often on the background of HBV- and HCV-related chronic disease (d'Arville *et al*, 1991; Nardone *et al*, 1996). In HCV-related cirrhosis, HCV replication was positively correlated with overexpression of IGF-2 (Tanaka *et al*, 1996).

We searched for morphologic correlates of deregulated IGF-2 and M6P/IGF-2R expression in conditions known to bear an increased risk for the development of HCC. Compared to architecturally normal liver, cirrhotic liver displayed reduced M6P/IGF-2R-specific staining of hepatocytes, whereas perisinusoidal cells showed increased immunoreactivity. Since this was a general finding, this staining pattern is unlikely to be explained by loss of an M6P/IGF-2R allele, previously demonstrated to occur in the vicinity of HCCs and dysplastic nodules (Yamada *et al*, 1997). Altered patterns of M6P/IGF-2R expression, as detected by the monoclonal antibody in our study, seem more likely to be related to altered gene regulation in the entire cirrhotic liver. This explanation is compatible with the observation of altered levels of IGF-2R in a rat model of hepatic carcinogenesis (Jirtle *et al*, 1994). In 11 of 18 cirrhotic livers, we observed clusters of hepatocytes overexpressing IGF-2, and a consistent decrease of hepatocellular M6P/IGF-2R-specific immunostaining in all of the examined cirrhotic liver specimens compared to normal liver indicating that these mechanisms play a role early in hepatocarcinogenesis prior to the appearance of dysplastic nodules. All of the 10 specimens with cirrhosis related to HBV or HCV infection were among those cases with clusters of hepatocytes overexpressing IGF-2, and altered M6P/IGF-2R staining patterns were common to all cirrhotic specimens. In one case, cholangiocytes also overexpressed IGF-2. This points to an involvement of the IGF-2 axis in cholangiocarcinogenesis as well. The staining patterns for IR and IGF-R1 as well as for IGFBP-3 and IGFBP-4, the former representing the dominant serum IGFBP activity also present in healthy liver (Wetterau *et al*, 1999), did not disclose significant differences between architecturally normal and cirrhotic livers. Immunostaining of sinusoidal cells with Kupffer cell morphology may represent phagocytosis of serum proteins including IGFBP by these cells. Thus, these observations do not argue against the hypothesis of a focally enhanced autocrine loop of IGF-2 acting back on hepatocytes expressing IGF-1R and IR. Determination of pericellular IGFBP levels in the microenvironment of hepatocytes overexpressing IGF-2 may be more informative in this respect.

In addition to the focal upregulation of IGF-2, we observed reduced M6P/IGF-2R-specific immunostaining in cirrhosis when compared to normal liver. The M6P/IGF2 receptor is involved in the transport of lysosomal enzymes and is considered a tumour suppressor gene by virtue of its capacity to bind, internalise, and degrade peptide growth factors including IGF-2 (De Souza *et al*, 1997). Reduced transcriptional activity of the M6P/IGF-2R promotor was observed in several human and rodent tumour cell lines (Jirtle *et al*, 1994). Moreover, loss of heterozygosity for M6P/IGF-2R was found with point mutations in the other allele in 70% of patients with HCC, and some of these mutations resulted in disruption of ligand binding (Byrd *et al*, 1999; Devi *et al*, 1999).

Whereas downregulation of M6P/IGF-2R appears as a diffuse process, upregulation of IGF-2 involves distinct foci of hepatocytes. All of this may result in an increased amount of free IGF-2, with a local oversaturation of the IGFBP-system and, ultimately, an increased stimulation of the IGF-1R. A relation between IGF-2 and cell proliferation was shown by Lin *et al* (1997), in the human hepatoma cell lines HuH-7 and HepG-2, both of which express IGF-2 at high levels. Treatment of these cell lines with IGF-2 antisense oligonucleotides resulted in reduced IGF-2 RNA and protein level as well as a reduced proliferative activity (Lin *et al*, 1997).

In conclusion, both focal upregulation of IGF-2 and general downregulation of M6P/IGF-2R seem to represent mechanisms operative early before the appearance of dysplastic changes in cirrhotic liver, particularly when related to chronic HBV and HCV infection. In different human osteosarcoma cell lines an inhibition of the IGF-1- and -2-stimulated uptake of thymidine as well as a partial inhibition of the basal DNA synthesis was observed when the IGF-1R was blocked by monoclonal antibody *α*-IR3 (Raile *et al*, 1994). Thus, raising the neutralisation capacities for IGF-2 in the space of Disse as well as blocking the IGF-1R may be interesting strategies to prevent the formation of HCC in patients at risk of developing this malignancy. Since hepatocytes overexpressing IGF-2 may be prone to acquire additional genetic changes, determination of hepatic IGF-2 levels and, in particular, morphologic assessment of IGF-2 overexpression may be indicators of an increased risk to develop HCC. The application of such techniques may be of interest to assess the individual risk for HCC in cases with HCV- and HBV-related chronic hepatitis and in cirrhosis of nonviral aetiology.
